# A fusion safety and security analysis framework for intelligent and connected vehicles

**DOI:** 10.1371/journal.pone.0332050

**Published:** 2025-09-22

**Authors:** Bin Sun, Shichun Yang, Yu Wang, Jiayi Lu, Zhaowen Pang, Xinjie Feng, Haoran Guang, Yaoguang Cao

**Affiliations:** 1 School of Transportation Science and Engineering, Beihang University, Beijing, China; 2 Innovation Center of New Energy Vehicle Digital Supervision Technology and Application for State Market Regulation, Beijing, China; 3 School of Information and Communication Engineering, Hainan University, Haikou, China; 4 State Key Lab of Intelligent Transportation System, Beihang University, Beijing, China; 5 Hangzhou International Innovation Institute, Beihang University, Hangzhou, China; Universita degli Studi della Campania Luigi Vanvitelli, ITALY

## Abstract

Driven by advancements in emerging technologies and data-driven innovations, the global automotive industry is focusing on intelligent and connected vehicles (ICVs), which involve complex electronic systems and vast data interactions. Safety concerns have expanded beyond traditional safety measures to include functional safety, safety of the intended functionality (SOTIF), and cybersecurity. Despite their interconnected nature, current methods often address these domains separately, risking incomplete safety assessments. This paper introduces a fusion safety analysis method that evaluates the three domains collectively. By identifying safety attributes and mapping unsafe behaviors to hazardous scenarios, it quantitatively assesses integrated safety risks. An illustrative case study on adaptive cruise control (ACC) highlights the method’s effectiveness, stressing the importance of addressing multi-dimensional safety issues to enhance ICVs safety.

## Introduction

With technological advancements in fields such as artificial intelligence (AI), fifth generation mobile communication technology (5G), and big data, intelligent and connected vehicles (ICVs) are becoming a focus in the global automotive industry [3]. Due to the extensive applications of these cutting-edge technologies, electronic systems in vehicles are becoming increasingly complex, and the volume of information interacting with vehicles is continuously growing. Therefore, the concept of safety for ICVs is no longer limited to traditional passive and active safety, but increasingly involves functional safety, the safety of the intended functionality (SOTIF), and cybersecurity [4], which constitute the three key safety domains in ICVs. Although each domain has its own unique focus, their coupled relationships and mutual influences cannot be ignored. However, most current methods address these safety concerns independently, which poses significant safety risks when confronted with the combined problems that involve the three types of safety. Therefore, a fusion safety analysis method is needed for systematic analysis and complete identification of fusion safety risks.

With the extensive applications of electronic control systems, such as the anti-lock brake system (ABS) and electronic stability program (ESP), the quantities and complexities of electronic components in automobiles have been continuously increasing. Consequently, the issue of functional safety in vehicles has begun to attract more attention. The ISO 26262 standard, developed from the International Electrotechnical Commission (IEC) 61508 standard, refers to the prevention of unreasonable risks caused by the malfunction of electronic and electrical systems [28].

The development of automotive intelligence has introduced emerging safety issues addressed by SOTIF. Unlike functional safety, which deals with functional failures, SOTIF focuses on safety issues arising from system performance limitations or reasonably foreseeable human misuse. The ISO 21448 standard has been published to regulate the development and design of SOTIF [29]. From a system engineering perspective, both functional safety and SOTIF are related to system safety, involving complex systems and interdisciplinary research. However, functional safety analyzes the safety risks caused by the unreliability of system, while SOTIF emphasizes the safety risks arising from limitations in system performance due to triggering conditions.

In addition to safety issues brought about by electrification and intelligence, security issues caused by connectivity have also gained widespread attention in the field of ICV safety. The rapid development of the Internet of Vehicles technology, while bringing convenience to system operations and enhancing the driver and passenger experience, also introduces cybersecurity risks such as cyberattacks and sensitive data leakage. ISO/SAE 21434 is the first international standard established for automotive cybersecurity, marking the advent of reliable cybersecurity mechanisms as an essential safety attribute for vehicles [30].

**Fig 1:**
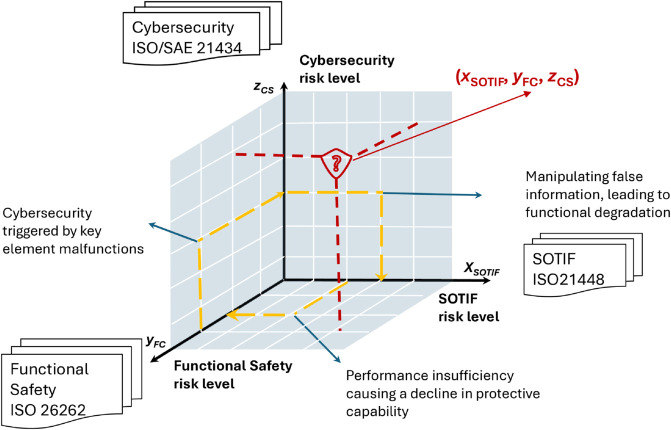
Interrelationships among functional safety, SOTIF, and cybersecurity. Illustration of the interrelationships among functional safety, SOTIF, and cybersecurity risk levels, forming a three-dimensional fusion safety space. Each axis corresponds to the risk level in one of the three safety domains: SOTIF (*x*_*SOTIF*_), functional safety (*y*_*fc*_),and cybersecurity (*z*_*CS*_). Yellow arrows indicate possible causal interactions among the domains: from functional safety failures leading to exploitable cybersecurity vulnerabilities, from cybersecurity threats manipulating sensor inputs and triggering SOTIF issues, and from SOTIF performance insufficiencies weakening functional safety capabilities. The red question mark at the risk point (*x*_*SOTIF*_, *y*_*FC*_, *z*_*CS*_) highlights the research gap in integrating risks across all three domains, underscoring the need for a comprehensive fusion safety framework in intelligent vehicle design.

Safety has always been a crucial topic in the development of the automotive industry, and the development of ICV technology has further extended the boundaries of safety to encompass security [1]. Currently, there are separate normative standards and recommended safety analysis methods for each of the three types of safety. However, considering the comprehensive impacts, the three safety issues show closely coupled characteristics. Fig 1 outlines the potential interactions between the three types of safety, adding complexity to the safety design of ICVs. A failure in functional safety can lead to the exposure of security vulnerabilities, triggering cybersecurity issues, as indicated by the yellow arrow from the y-axis to the z-axis. For example, in August 2023, Oleg Drokin’s team at the Technical University of Berlin successfully gained unrestricted access to Tesla vehicles by exploiting a third-generation media control unit (MCU-Z) flaw using a voltage fault injection attack [17]. is becoming increasingly important, as it addresses security threats leading to physical hazardous scenarios [31]. SOTIF also needs to be considered within the cybersecurity framework, as the yellow arrow from the z-axis to the y-axis. Zhang et al. evaluated the impact of different adversarial cyber attacks on the detection accuracy of deep learning-based 3D object detection models [27]. In contrast, SOTIF involves new technologies such as AI and machine learning, which may introduce new functional safety and cybersecurity risks. The realization of the intended functionality of the system depends on the correct operation of these new technologies, and their introduction increases the general uncertainty of the system [6]. The yellow arrow from x-axis to y-axis illustrates the interrelationship.

Although several thorough safety analysis approaches have been developed across various safety domains, they usually focus on only one or, at most, two domains and lack a unified, systematic framework for all three safety aspects. These isolated methods are insufficient to address the increasingly complex safety requirements of ICVs, as overlooking any safety aspect poses significant risks. To overcome this limitation, this article proposes a fusion ICVs safety risk analysis method with a systematic framework that comprehensively analyzes the integration of the three safety domains. The fusion analysis method identifies the fusion safety-critical systems, constructs the mapping relationship between unsafe behaviors and hazardous scenarios, and quantitatively assesses the fusion safety risk value. This method represents the first attempt to effectively analyze all three types of safety within a unified framework, addressing the current fragmentation of safety domains. It provides an important tool for the future design of ICVs.

The remainder of this paper is organized as follows. The **Review of existing integrated analysis method** section reviews current integrated safety and security analysis methods. The **Proposed fusion safety analysis approach** section introduces the proposed fusion safety analysis method. The **Case study** presents an illustrative analysis case using the proposed method. The **Discussion** section provides further analysis, and the **Conclusion** section summarizes the entire paper.

## Review of existing integrated analysis method

The safety design of the systems is crucial to ensuring vehicle safety. During the system design phase, it is essential to employ a systematic and scientific safety analysis method to identify potential safety risks, establish corresponding safety requirements, and develop risk mitigation measures. The widespread application of AI and connected technologies has resulted in a deep integration of vehicle software and hardware, leading to increased attention to the extensive safety challenges faced by ICVs. In recent years, researchers have begun to explore these intersecting safety issues.

Research on the integrated analysis of functional safety and SOTIF includes the following. Kirovskii and Gorelov [8] merged the functional safety and SOTIF lifecycles by introducing specific requirements and SOTIF methods into the functional safety lifecycle. Kramer et al. [9] proposed a comprehensive method to evaluate the safety of autonomous driving systems, integrating identified issues of functional insufficiency or failure into the analysis of incorrect behavior for each functional unit. Zeller [10] introduced the concept of component fault and deficiency tree (CFDT) by extending the component fault tree (CFT) method, attributing functional safety and SOTIF issues to abnormal causes in certain components and analyzing them as root events in a logical tree. Although the aforementioned methods have made initial attempts to integrate functional safety and SOTIF, this integration typically remains confined to individual aspects, such as safety requirements or hazard identification, lacking a comprehensive, systematic quantitative analysis approach. Furthermore, these methods often overlook the impact of cybersecurity on safety.

Some reports on the integrated analysis of functional safety and cybersecurity issues are listed below. Amorim et al. [11] described a systematic development process that continuously iterated and updated the impacts of new safety requirements on existing safety analyses, ensuring that all functional safety and cybersecurity requirements were met. Triginer et al. [13] combined system theory methods with reliability theory methods to obtain a list of common requirements. They used STPA to identify unsafe and insecure actions, analyzing safety and security risks separately to establish the corresponding safety constraints. However, the above methods lack a quantitative evaluation and prioritization of the safety requirements. Wolf [12] mapped cybersecurity threats to vehicle failures and assessed the level of threat by calculating the impacts of these failures. Agrawal et al. [14] proposed an integrated framework called THARA to unify functional safety and cybersecurity. This framework reflects the impact of cybersecurity on functional safety in terms of risk controllability, serving as a constraint to address functional safety risks. It also considers the impact of functional safety on cybersecurity by incorporating functional safety risks into the severity of security risks, thereby integrating these risks into the overall assessment of cybersecurity risks for the system. Menekse and Tinaz [15] analyzed the potential risk levels caused by cybersecurity and functional safety in different scenarios for the same use case. Khatun et al. [16] extended the scenario-based HARA analysis method proposed in 2021 to a scenario-based TARA analysis, with the aim of identifying and analyzing potential hazards in the system and their vulnerability to cybersecurity attacks. Although there has been some progress in the integration of functional safety and cybersecurity methods, a research gap persists in unifying cybersecurity with SOTIF methods. Cyberattacks can manipulate sensor data, such as artificially adding noise to images to cause visual sensor recognition anomalies, which can lead to SOTIF issues for vehicles. The insufficient performance of system design may increase the system’s vulnerability to cyber threats.

The approaches reviewed above mainly focus on the interactions between two types of safety, with limited consideration of other dimensions. Although Kaneko et al. [19] proposed a method for the integrated management of functional safety, SOTIF, and cybersecurity standards, they highlighted that HARA and TARA cannot be simply merged due to differences in granularity and focus. However, they did not provide specific design methods. A quantitative, comprehensive analysis method that effectively tackles all three types of safety issues is currently absent. Our review of the literature reveals a significant research gap in the comprehensive analysis of these three safety domains.

In summary, current integrated analysis methods primarily focus on limited aspects of comprehensive analysis, such as safety requirements, controllability, and others, and remain in the early stages of development. Moreover, these methods mainly rely on subjective evaluation, which lacks quantification and prevents effective comparisons across different systems, subfunctions, or components. As a result, they are not capable of addressing the full spectrum of safety challenges in intelligent and connected systems.

In contrast, our proposed method addresses these limitations by offering a comprehensive, systematic, and quantifiable framework that integrates functional safety, SOTIF, and cybersecurity. To contextualize its contributions, we compare it with representative safety analysis methods—STPA, CFDT, and THARA—and highlight the key methodological innovations. STPA focuses on system-level causal reasoning but lacks quantitative severity evaluation; our method complements this by introducing a structured risk matrix to derive measurable fusion risk values. CFDT models fault propagation via fault trees, whereas we unify faults, performance insufficiencies, and cyberattacks into a single cross-domain risk model. Unlike THARA, which qualitatively links functional safety and cybersecurity, our approach quantitatively captures multi-domain interactions through gain factors for system interdependence and human misuse, enabling scalable and comparative assessments. By addressing the full spectrum of safety concerns and enabling objective, cross-system comparisons, our method reduces reliance on subjective judgment and offers a more robust, extensible, and practical solution to the evolving safety challenges in intelligent vehicle systems.

## Proposed fusion safety analysis approach

**Fig 2:**
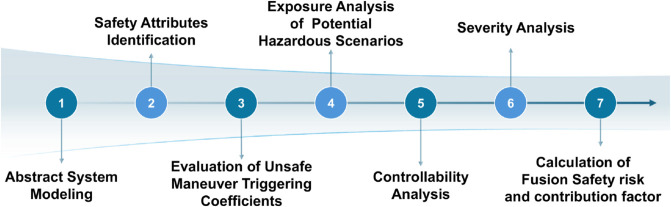
Overview of the proposed fusion safety analysis approach. The framework integrates seven sequential steps, from system modeling and attribute identification to risk calculation, providing a structured method for evaluating safety and security.

To bridge the existing methodological gap and systematically analyze the intertwined challenges of functional safety, SOTIF, and cybersecurity, we propose a structured fusion safety analysis framework. Fig 2 presents a schematic overview of the seven-step process, offering readers a visual roadmap of the proposed analytical flow.

The system is abstractly modeled to clarify internal hierarchies and external interactions based on specifications and definitions. By identifying integrated safety attributes, the priority of safety risk analysis for ICVs is established, focusing first on critical systems. The triggering factors of unsafe maneuvers are then assessed at the vehicle level. A correlation is established between unsafe maneuvers and hazardous scenarios, requiring analysis of each scenario’s exposure rate. In these scenarios, the controllability of the safety risks and the severity of potential accidents are analyzed. The fusion safety risk value is calculated by combining the triggering coefficient, controllability, exposure, and severity. Finally, the contribution of different risk factors to the overall risk is determined, identifying the vulnerability of the system and guiding optimization efforts.

Furthermore, the overall structure of the proposed fusion safety analysis framework is inherently compatible with the ISO 26262-based V-model development process. The step-by-step analytical workflow—from system abstraction to safety attribute identification, hazardous scenario mapping, and fusion risk quantification—can be conducted in parallel with the V-cycle activities of functional safety and SOTIF. For example, fusion risk identification and prioritization can support safety goal definition and technical safety requirement refinement during the concept and system design phases. This seamless alignment enables the proposed framework to serve as a cross-domain risk analysis layer that enhances conventional safety engineering practices without disrupting existing workflows.

### Abstract system modeling

**Fig 3:**
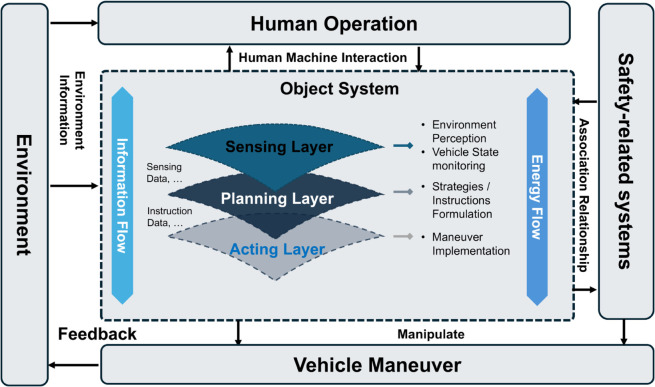
Schematic of abstract system modeling. The model depicts an object system that interacts with the environment, human operators, and safety-related systems, and is internally structured around three layers—sensing, planning, and acting—linked by information and energy flows.

Abstract modeling simplifies a system’s internal complex functional logic into three functional layers: sensing, planning, and acting, along with the interlayer interactions of information and energy flow, as shown in Fig 3. This modeling approach is flexible, allowing component adjustments based on actual conditions, so we can comprehensively understand the system external interaction relationships while considering the impacts of the external environment, systems, and human operations on the system. It provides a foundation for the design and analysis of ICV systems.

The sensing layer involves information acquisition, such as environmental perception and vehicle state monitoring, using sensors such as cameras and radars. The planning layer formulates strategies and produces information-based instructions, using hardware such as ECUs and decision-making algorithms. The acting layer executes actions through controllers and actuators. Information flow involves the exchange of data (e.g., sensing information and operating instructions) between layers, whereas energy flow refers to energy exchange between components, such as mechanical energy transmitted through transmission mechanisms and hydraulic pipelines.

In modeling external interactions, human-machine interaction, external system impact, and the effects of system output on vehicle behavior are considered. Analyzing safety-related systems, like the dependency of autonomous emergency braking on the braking system’s normal operation, is also crucial.

Specifically, let *A* represent the set of all possible vehicle maneuvers that the studied system can influence, such as acceleration, deceleration, or lane change. Define unsafe vehicle maneuvers as those that can cause accidents or pose a threat to the safety of passengers, other road users, and the environment. Use $A_{hazardous}\in A$ to represent the set of all unsafe vehicle maneuvers and ${a_{hazardous,}}_{i}\in A$ to represent a specific unsafe maneuver. Therefore, the set of unsafe vehicle maneuvers can be expressed as ${A_{hazardou}}_{s}=\{a_{1},a_{2}\cdots a_{k}\}$ where *k* denotes the total number of hazardous maneuvers. Thus, the set of unsafe vehicle maneuvers can be defined as

$$A_{\text{hazardous}}=\left\{a_{\text{hazardous}}\in A|a_{\text{hazardous}}\text{ may cause accidents or threaten safety}\right\}.$$ (1)

### Safety attributes identification

Following the procedure above, we have clarified the internal functional logic and the external interaction relationships of the system. In this section, we quantify the system’s integrated safety attributes based on its internal functional logic relationships. This allows us to determine the priorities for the integrated safety risk analysis for ICVs, ensuring that the critical integrated safety elements are analyzed and evaluated first. This process is divided into four subprocesses: functional safety attribute analysis, SOTIF attribute analysis, cybersecurity attribute analysis, and fusion safety attribute evaluation.

#### Functional safety attribute analysis.

Based on the established abstract modeling of the research system, we analyze the specific functional elements at each level, including the sensing layer, the planning layer, and the acting layer. We define the system function set as *Function*_*layer*_ where layer∈{sensing,planning,acting}. According to this *Function*_*layer*_, we identify all possible failure modes and denote them by *Fault*_*layer*_. Let *fault*_*j*_ be a failure mode of the specific function, then $fault_{j}\in Fault_{layer}$ and $⋃fault_{j}=Fault_{layer}$. Through analysis, we establish the mapping relationship between *fault*_*j*_ and an unsafe vehicle maneuver:

$$y^{fault}=f(fault_{j},a_{\text{hazardous},i}),\;y^{fault}\in \{0,1\},$$ (2)

where *y^fault^* represents the analysis result. If the failure of a functional item *fault*_*j*_ directly leads to unsafe vehicle behavior, then record *y^fault^* as 1; otherwise, it is 0. *m* and *n* represent the total number of unsafe maneuvers and failures, respectively. Based on the analysis, the functional safety matrix *M*_*FC*_ is expressed as follows:

$$M_{FC}=[\begin{array}{cccc} y_{1,1}^{fault} & y_{1,2}^{fault} & \cdots & y_{1,n}^{fault}\\ y_{2,1}^{fault} & y_{2,2}^{fault} & \cdots & y_{2,n}^{fault}\\ \vdots & \vdots & \ddots & \vdots \\ y_{m,1}^{fault} & y_{m,2}^{fault} & \cdots & y_{m,n}^{fault} \end{array}]=(y_{i,j}^{fault}{)}_{m\times n},$$ (3)

where $y_{i,j}^{fault}$ represents the Boolean calculation result of the *j*-th functional failure leading to the *i*-th unsafe maneuver. The system’s functional safety attribute value $V_{FC}$ is determined based on the Frobenius norm of *M*_*FC*_:

$$V_{FC}={\left\|M_{FC}\right\|}_{F}.$$ (4)

#### SOTIF attribute analysis.

Unlike functional safety, the SOTIF issue refers to a situation where neither software nor hardware fails but the system fails to avoid safety risks under certain triggering conditions. According to ISO 21448’s definition of insufficiency, an output insufficiency, either by itself or in combination with one or more output insufficiencies of other elements, contributes to a hazardous behavior at the vehicle level [29]. Therefore, in this text, we consider the collection of outputs as *Output*_*layer*_, where layer∈{sensing,planning,acting}. Let the output insufficiency at the layer level be *Insufficiency*_*layer*_, and let the insufficiency of a specific output be *insufficiency*_*k*_, then $insufficiency_{k}\in Insufficiency_{layer}$, and $⋃insufficiency_{k}=Insufficiency_{layer}$.

Through analysis, we establish the mapping relationship between *insufficiency*_*k*_ and unsafe vehicle behaviors as defined by the following function:

$$y^{ins}=h({\text{insufficiency}}_{k},a_{\text{hazardous},i}),\;y^{ins}\in \{0,1\}.$$ (5)

The results of the analysis are expressed in Boolean form. If insufficiency directly leads to unsafe vehicle behavior, then *y^ins^* is recorded as 1; otherwise, it is 0. If there is a total of *q* insufficiencies, the SOTIF matrix *M*_*SOTIF*_ is expressed as follows:

$$M_{sotif}=[\begin{array}{cccc} y_{1,1}^{ins} & y_{1,2}^{ins} & \cdots & y_{1,q}^{ins}\\ y_{2,1}^{ins} & y_{2,2}^{ins} & \cdots & y_{2,q}^{ins}\\ \vdots & \vdots & \ddots & \vdots \\ y_{m,1}^{ins} & y_{m,2}^{ins} & \cdots & y_{m,q}^{ins} \end{array}]=(y_{i,k}^{ins}{)}_{m\times q},$$ (6)

where $y_{i,k}^{ins}$ represents the Boolean calculation result of the *k*-th insufficiency and the *i*-th unsafe maneuver. The SOTIF attribute value $V_{SOTIF}$ is determined based on the Frobenius norm *M*_*sotif*_, calculated using the following formula:

$$V_{SOTIF}={\left\|M_{sotif}\right\|}_{F}.$$ (7)

#### Cybersecurity attribute analysis.

First, analyze the internal interactions of the system. If there is only energy flow without information interaction, cybersecurity attribute analysis is not required, and the cybersecurity attribute value $V_{SI}=0$. Otherwise, all information flows existing between the system layers should be identified and defined as *Inf*. For each information flow, define *inf*_*flow*,*l*_, $inf_{flow,l}\in Inf$. The threat-based method in cybersecurity field can be applied to identify potential cyber threats, denoted as *cs*_*threat*,*u*_. A cyber threat set *CS*_*threat*_ that includes all identified cyber threats is constructed, so $cs_{threat,u}\in CS_{threat}$, and $⋃cs_{threat,u}=CS_{threat}$.

Subsequently, we analyze the cybersecurity attributes of the information flow that can be compromised in network threat scenarios, establishing a mapping relationship between each type of threat, the information flow, and functional failures or insufficiencies.

The mapping relationship can be expressed as:

$$z_{v}=g(inf_{flow,l},cs_{threat,u}),$$ (8)

where $z_{v}\in Z=\{z_{1},z_{2},\cdots ,z_{w}|z\in Fault_{layer}\cup Insufficiency_{layer},\;w\leq m+n\}$, *Z* represents the set of mapped functional failures or output insufficiencies. For example, if a spoofed controller area network (CAN) message is sent to the brake’s ECU, it may result in a functional failure of the braking system. If $z_{v}\in Fault_{layer}$, substitute it into Eq (2) to calculate the Boolean result. Otherwise, if $z_{v}\in Insufficiency_{layer}$, substitute it into Eq (5). The calculation results are shown in Eq (9).

$$y^{inf}=\left\{ \begin{array}{cc} f(z_{v},a_{i}), & z_{v}\in Fault_{layer}\\ h(z_{v},a_{i}), & z_{v}\in Insufficiency_{layer} \end{array}\right..$$ (9)

Similarly, based on the calculation results, the cybersecurity matrix *M*_*CS*_ is expressed as follows:

$$M_{CS}=[\begin{array}{cccc} y_{1,1}^{inf} & y_{1,2}^{inf} & \cdots & y_{1,p}^{inf}\\ y_{2,1}^{inf} & y_{2,2}^{inf} & \cdots & y_{2,p}^{inf}\\ \vdots & \vdots & \ddots & \vdots \\ y_{m,1}^{inf} & y_{m,2}^{inf} & \cdots & y_{m,p}^{inf} \end{array}]=(y_{i,l}^{inf}{)}_{m\times p}.$$ (10)

Here $y_{i,l}^{\text{inf}}$ represents the Boolean calculation result of the *l* th information flow and the *i* th unsafe maneuver. *p* represents the total number of information flows in a system.

The system’s cybersecurity attribute value is determined based on the Frobenius norm of *M*_*CS*_, calculated using the following formula:

$$V_{CS}={\left\|M_{CS}\right\|}_{F}.$$ (11)

#### Fusion safety attribute evaluation.

By analyzing the system functional safety, SOTIF, and cybersecurity attributes of the system, we obtain the functional safety analysis matrix *M*_*FC*_ and its safety attribute value $V_{FC}$, the SOTIF matrix *M*_*SOTIF*_ and its safety attribute value $V_{SOTIF}$, and the cybersecurity analysis matrix *M*_*CS*_ and its safety attribute value $V_{CS}$. These three safety matrices are then combined to obtain the final fusion safety attribute analysis matrix:

$$M_{FS}=[M_{FC}|M_{SOTIF}|M_{CS}].$$ (12)

The fusion safety attribute value is calculated based on Eq (12) by

$$V_{FS}=\lambda \cdot {\left\|M_{FS}\right\|}_{F},$$ (13)

with

$$\lambda =\text{sgn}(V_{FC})+\text{sgn}(V_{SI})+\text{sgn}(V_{CS})-\max[\text{sgn}(V_{FC}),\text{sgn}(V_{SI}),\text{sgn}(V_{CS})].$$ (14)

Here, the fusion factor *λ* serves as a domain interaction indicator that scales the overall fusion safety value $V_{FS}$, capturing the compounded effect of multi-domain safety issues. It increases as more safety domains—functional safety, SOTIF, and cybersecurity—simultaneously contribute to the overall risk. Specifically, *λ* equals 0 when only one domain is involved, 1 when two domains contribute, and 2 when all three domains are active. This formulation reflects the escalating complexity and integration challenges associated with cross-domain safety interactions.

### Evaluation of unsafe maneuver triggering coefficients

From the fusion safety risk analysis matrix *M*_*FS*_ obtained above, we map functional failures, performance insufficiencies, and cyber threats to unsafe vehicle behaviors. At the level of unsafe maneuvers, we conduct a comprehensive analysis of the impacts of these three safety issues. In other words, unsafe vehicle behaviors result from the compounded state of functional safety, SOTIF, and cybersecurity issues.

In this step, we calculate the triggering risk coefficients for the three safety issues and derive the integrated safety risk triggering coefficient. When assessing the risk of unsafe vehicle behaviors triggered by functional safety issues, we evaluate each functional item layer by layer. By analyzing statistical data and calculating the failure rate of each functional element of the system, we denote the failure rate of the *j* -th functional element as *p*_*FC*,*j*_.

To better handle the failure rates of the system components that may vary significantly and span multiple orders of magnitude, we logarithmically transform the failure rates and compress these differences into a more manageable range. We construct the functional safety risk triggering coefficient vector for the entire system as follows:

$$P_{FC}=[\begin{array}{c} \ln(p_{FC,1})+b\\ \ln(p_{FC,2})+b\\ \vdots \\ \ln(p_{FC,n})+b \end{array}],$$ (15)

where $b\geq \left|\min[\ln(p_{FC,1}),\ln(p_{FC,2}),\ldots ,\ln(p_{FC,m})]\right|$is the evaluation adjustment coefficient. Since the logarithmic function produces negative values when handling numbers less than 1, the positive constant *b* is added to ensure positive results, facilitating subsequent processing.

When assessing the risk of unsafe vehicle behaviors triggered by SOTIF issues, it is necessary to evaluate each output item layer by layer, analyze the triggering conditions that cause output insufficiencies, and determine the probability that each triggering condition will lead to a system performance insufficiency. For the *k*-th insufficiency, the overall triggering probability is calculated, denoted *p*_*SOTIF*,*j*_, which directly results from the occurrence rate of the triggering conditions that can activate the functional insufficiencies leading to hazardous maneuvers [29]. Similarly, the vector of the SOTIF risk triggering coefficient for the entire system is constructed as follows:

$$P_{SOTIF}=[\begin{array}{c} \ln(p_{SOTIF,1})+b\\ \ln(p_{SOTIF,2})+b\\ \vdots \\ \ln(p_{SOTIF,q})+b \end{array}].$$ (16)

When assessing the risk of unsafe vehicle behaviors triggered by cybersecurity issues, we evaluate each information flow *inf*_*flow*,*l*_ by analyzing the feasibility of cyberattacks to obtain the system vulnerability assessment score $vul_{l}$. The assessment of system vulnerability refers to the methods suggested by ISO 21434. Using these scores, we construct the vector of the cybersecurity risk triggering coefficient for the entire system as follows:

$$VUL=[\begin{array}{c} vul_{1}\\ vul_{2}\\ \vdots \\ vul_{p} \end{array}].$$ (17)

We believe that functional failures and performance insufficiencies are inherent system properties that lead to unsafe vehicle behaviors, while system vulnerabilities to threats stem from external sources. Therefore, when calculating the trigger coefficient for unsafe behaviors, the vulnerability of the system is considered a multiplicative factor based on the original foundation of the system. With the triggering coefficient vectors $P_{FC},P_{SOTIF},VUL$ obtained from the analysis above, we calculate the composite triggering coefficient for unsafe maneuvers, which results from multiple overlapping safety problems. The triggering coefficient for the *i*-th unsafe maneuver is calculated by

$$F_{i}=\left\{ \begin{array}{cc} \left[(M_{FC}{)}_{i,*}\cdot P_{FC}+(M_{sotif}{)}_{i,*}\cdot P_{sotif}\right]\cdot \left[(M_{CS}{)}_{i,*}\cdot VUL\right], & \|(M_{CS}{)}_{i,*}\|\neq 0\\ \left[(M_{FC}{)}_{i,*}\cdot P_{FC}+(M_{sotif}{)}_{i,*}\cdot P_{sotif}\right], & \|(M_{CS}{)}_{i,*}\|=0 \end{array}\right..$$ (18)

The triggering coefficient vector for the system overall unsafe vehicle behaviors is:

$$F_{FS}=[\begin{array}{cccc} F_{1} & F_{2} & \cdots & F_{m} \end{array}{]}^{T},$$ (19)

where *m* represents the total number of triggering coefficients for unsafe maneuvers.

### Exposure analysis of potential hazardous scenario

The ISO 21434 and ISO 21448 standards emphasize that safety analysis involves calculating the exposure by assessing potential hazardous scenarios that could result in hazardous events. For example, if an ICV incorrectly identifies a bridge ahead as an obstacle and performs unexpected braking, a collision accident will only occur if another vehicle follows it. This means that danger typically only causes harm in specific scenarios. Therefore, for each unsafe maneuver, it is necessary to consider its associated hazardous scenarios to complete the exposure rate analysis, denoted as *e*_*i*,*t*_. Here, *i* represents the corresponding unsafe maneuver and t represents the hazardous scenario associated with that unsafe maneuver.

ISO 26262-3 provides an exposure evaluation method that estimates the exposure rate of potentially hazardous scenarios in two ways. The first is based on the duration of a specific hazardous scenario, and the second is based on the probability of encountering a hazardous scenario. For example, the exposure rate can be estimated using the average time that a vehicle passes through an intersection on the road. It can also be estimated using the frequency with which the vehicle passes through the same road intersection. The exposure, based on a representative sample of operating scenarios in the target market, can be categorized into four levels, from E1 to E4, according to the two estimation approaches mentioned above. It is important to note that if driving conditions allow the use of both exposure rate evaluation approaches but yield different results, a detailed analysis is required to select the more appropriate approach.

### Controllability analysis

Controllability serves as an indicator to evaluate the capability to manage risks within identified hazardous scenarios. According to the definition in ISO 26262, a higher controllability assessment value indicates poorer risk manageability. However, this standard predominantly considers human intervention-based risk control capabilities, overlooking the inherent risk control capabilities that advanced autonomous driving systems should possess. In addition, considerations regarding human factors tend to neglect the impact of human error. Therefore, within the framework proposed in this paper, we extend the traditional notion of controllability beyond human intervention alone to encompass the inherent risk control capabilities of the system. Rating scores and their criterion are listed in detail [5,7]. Furthermore, when assessing controllability regarding human and system aspects, it is crucial to account for the influence of human misuse and analyze how other associated systems affect the system under study.

In the framework proposed in this article, the modified controllability is adjusted using the controllability gain factor $\beta_{safety}$ and the gain factor for human misuse $\beta_{misuse}$. In addition, a scoring-based quantitative evaluation method is introduced to calculate these factors. It should be noted that when applying this framework for integrated safety analysis, other reasonable methods can also be employed to assess human misuse and the impacts of related systems.

To assess $\beta_{safety}$, the analysis focuses on the relationships between the target system and other safety-related systems. It involves identifying categories of system interdependencies, including supportive, redundant, and protective relationships. Supportive relationships denote the instances where the safety functions of the target system rely on basic functionalities of other systems, such as AEB depending on the normal operation of the braking system. Redundant relationships refer to backup systems beyond the inherent redundancy of the target system, which can improve reliability in the event of safety risks. Protective relationships involve additional safety systems beyond the inherent protective capabilities of the target system, such as a “safety copilot system”.

Based on the analysis of the interdependencies of safety functions between systems, the system controllability gain factor $\beta_{safety}$ is obtained as

$$\beta_{safety}=1+\log\left(\frac{\beta_{SUP}}{\beta_{RED}\cdot \beta_{PRO}}\right).$$ (20)

To assess the human misuse gain factor $\beta_{misuse}$, one needs to analyze the types of interactions between the human driver and the system, categorizing them into information and action types. Information types generally include visual alert messages and auditory reminders, while action types involve actions such as taking control of the steering wheel or pressing the brake pedal. Specifically, the Interaction Information factor ($\beta_{\text{Inf}}$) is intended to reflect the interface usability and driver cognitive load—a higher score indicates that the interface is harder to interpret or more mentally demanding to process. Meanwhile, the Interaction Operation factor ($\beta_{\text{OP}}$) captures the complexity and physical demand of the required driver response, which is closely related to user response time and action execution difficulty. Together, these two components allow the framework to quantify the human-system interaction burden in a structured way, and provide a foundation for extending controllability analysis in increasingly automated driving environments.$\beta_{misuse}$ is then calculated based on these two factors, as given in Eq (21).

$$\beta_{misuse}=1+\log(\beta_{Inf}\cdot \beta_{OP}).$$ (21)

The assessment scores for the system interdependency impact factors and human misuse are detailed in Table 1.

**Table 1:** Impact assessment scores for system interdependency and human misuse. This table outlines the evaluation criteria for assessing system interdependency, focusing on the aspects of support, redundancy, and protection, as well as human misuse, which is analyzed through interaction information and operation.

Type Score	1	2	3
**Support $\beta_{\text{SUP}}$**	**The system operates completely independently without relying on other systems.**	Moderate supportive relationship: The system depends on some other systems, but failures in these systems do not lead to major functional failures.	Highly supportive relationship: The system depends on multiple other systems. Failures in these systems lead to major failures.
**Redundancy $\beta_{\text{RED}}$**	There are no additional redundant systems.	Additional redundant systems provide partial functionality.	Additional redundant systems provide full functionality.
**Protection $\beta_{\text{PRO}}$**	There are no additional protective systems.	Additional protective systems only offer basic functions such as monitoring and alerting.	Additional protective systems can execute emergency evasion responses in critical situations.
**Interaction Information $\beta_{\text{Inf}}$**	The information is easy to understand and is presented in a straightforward and clear manner.	The information is somewhat challenging and requires a detailed explanation of several steps.	The information is complex, involving technical jargon and specialized concepts.
**Interaction Operation $\beta_{\text{OP}}$**	The operation is simple and requires no additional explanation, such as when it involves only a single straightforward step.	The operation involves some complexity and requires corresponding operational guidance, such as selecting from different options or understanding basic rules and procedures.	The operation is complex, requiring users to invest significant time in learning and adapting, such as a task that involves making decisions across multiple stages.

The modified controllability is calculated by

$$c_{i,t}=\left\{ \begin{array}{cc} \beta_{safety}\cdot c^{sys}, & \text{if }c^{sys}\in \{c1,c2\}\\ \beta_{safety}\cdot c^{sys}+\beta_{misuse}\cdot c^{D}, & \text{otherwise} \end{array}\right.,$$ (22)

where *i* denotes the *i*-th unsafe maneuver, and *t* denotes the *t*-th scenario associated with the *i*-th unsafe maneuver.

### Severity analysis

The severity of potential harm shall be estimated based on a defined rationale for each hazardous event. Referring to the severity classes in ISO 26262-3, abbreviated injury scale (AIS) classification is employed to categorize injury severity levels denoted by *s*_*i*,*t*_, ranging from S0 to S3.

### Calculation of fusion safety risk and contribution factor

Based on the unsafe triggering coefficient, exposure, controllability, and severity, the fusion safety value for different safety behaviors is calculated by

$$risk_{i}=\sum_{t}(F_{FS}{)}_{i}\cdot e_{i,t}\cdot c_{i,t}\cdot s_{i,t},$$ (23)

where *F*_*FS*_ is the triggering coefficient vector, *e*_*i*,*t*_ represents controllability in the *t*-th hazardous scenario, *c*_*i*,*t*_ denotes the exposure in the *t*-th hazardous scenario, and *s*_*i*,*t*_ signifies the severity in the *t*-th hazardous scenario.

The total fusion safety risk value is calculated as:

$$risk_{FS}=\sum_{i}risk_{i}.$$ (24)

By employing the partial differentiation chain rule for backtracing, the safety risk contribution factor (RCF) for each factor is computed to identify the factor that has the most substantial impact on the fusion safety risk value. The RCF indicates how each factor influences the output, thereby prioritizing directions for improvement. The calculation formula is as follows:

$${\text{RCF}}_{fault,j}=p_{FC,j}\cdot \frac{\partial risk_{FS}}{\partial p_{fault,j}}=p_{FC,j}\cdot \sum_{i=1}^{m}\frac{\partial risk_{i}}{\partial F_{i}}\cdot \frac{\partial F_{i}}{\partial p_{FC,j}},$$ (25)

$${\text{RCF}}_{insufficiency,k}=p_{SOTIF,k}\cdot \frac{\partial risk_{FS}}{\partial p_{insufficiency,k}}=p_{SOTIF,k}\cdot \sum_{i=1}^{m}\frac{\partial risk_{i}}{\partial F_{i}}\cdot \frac{\partial F_{i}}{\partial p_{SOTIF,k}}.$$ (26)

It is worth noting that since we consider threats as external stimuli and map them to corresponding functional failures or performance insufficiencies, the calculations for each factor above already include the impact of information threats. Consequently, to demonstrate the influence of cyberattacks on the contribution factor, we adopt an alternative calculation method. In the overall safety risk value, we differentiate between the safety risks not influenced by network threats and those influenced by network threats. Though assessing the risk contribution of cybersecurity, here we calculate the contribution of a specific network threat to precisely identify threat scenarios and effectively enhance the system’s resilience:

$${\text{RCF}}_{threat,l}=\frac{\partial risk_{FS}}{\partial vul_{l}}=\sum_{i=1}^{m}\frac{\partial risk_{i}}{\partial F_{i}}\cdot \frac{\partial F_{i}}{\partial vul_{l}}.$$ (27)

## Case study

In this section, we analyze a hypothetical ACC system to demonstrate the effectiveness of the proposed method. The system uses drive-by-wire technology, controlling braking and acceleration through electronic signals sent via wires to actuators, rather than relying on mechanical linkages or hydraulic fluids. Its drive-by-wire architecture, which reduces mechanical complexity and heightens cybersecurity concerns, makes it an ideal example for showcasing multi-domain safety analysis.

### Abstract system modeling

First, the ACC system architecture is further abstracted and modeled [22], which is divided into the sensing, planning, and acting layers. The sensing layer recognizes the targets ahead of the vehicle, gathers vehicle status, and transmits these results to the planning layer. To determine the status of the vehicle, it is essential to gather data on the brake pedal condition as well as the vehicle’s speed. The planning layer primarily verifies the system’s status and decides whether to maintain the set speed, decelerate, or restore to the set speed based on the target status collected by the sensing layer. The planning layer controls the brake system and power system separately according to the instructions from the planning layer.

**Fig 4:**
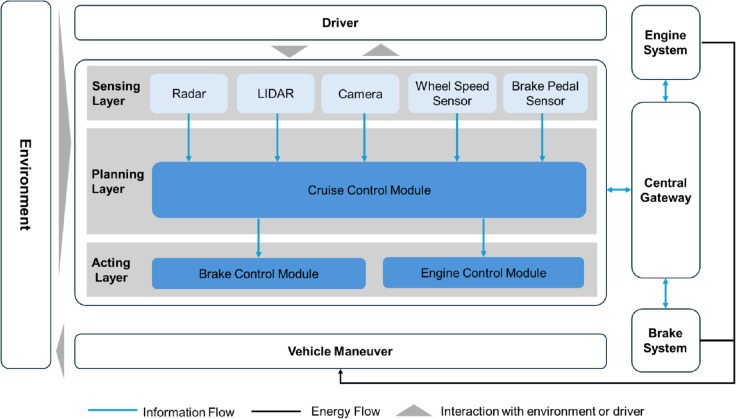
Schematic of abstract modeling in the case study. Based on the abstract system modeling, the figure illustrates the architecture of an adaptive cruise control (ACC) system, including sensing, planning, and acting layers, and their interactions with the driver, environment, and vehicle systems.

The hypothetical ACC external associated systems comprise the central gateway, the power system, and the brake system. The ACC functions by transmitting operational signals to the central gateway, which redirects them to designated ECUs for vehicle behavior control. The central gateway, brake system, and power system are considered dependent subsystems in the ACC system, crucial for the proper functioning of ACC operations. The ACC system discussed adjusts vehicle speed and adapts to road conditions automatically based on set parameters, maintaining safe distances from preceding vehicles or other traffic participants. Fig 4 depicts the abstract model. This system manages only the longitudinal movement of the vehicle. Therefore, according to Eq (1), unsafe maneuvers arising from this system are expressed by $A_{hazardous}=\{a_{hazardous,1},a_{hazardous,2},a_{hazardous,3}\}$, where *a*_*hazardous*,1_, *a*_*hazardous*,2_, and *a*_*hazardous*,3_ denote the unintended acceleration, unintended deceleration, and unintended speed maintenance, respectively.

### Fusion safety attribute identification

To provide a more intuitive representation of the analysis process, we select several key functions of the ACC for step-by-step analysis, rather than analyzing all function failures.

#### Functional safety attribute.

In this context, we utilize the HAZOP analysis method and apply a guide word-driven strategy to examine functional failure modes within various layers. Here, we focus on typical failure modes of the planning and acting layers.

At the planning level, a set of failure modes is expressed through the HAZOP process as $Function_{plan}=\{fault_{1},fault_{2}\}$, where *fault*_1_ means “No activation of the ACC when the ON signal is received”, and *fault*_2_ represents “No deactivation of the ACC when the OFF signal is receive”. For the acting layer, $Function_{act}=\{fault_{3},fault_{4}\}$, where *fault*_3_ means “More acceleration after the default speed is reached”, and *fault*_4_ represents “Less acceleration than necessary to reach the default speed”. To identify the functional relationship expressed in Eq (2), we employ an expert method to determine the connections between functional failures and unsafe vehicle maneuvers, establishing their mapping relationship as shown in S1 Table (a). It is worth noting that the relationship expressed in Eq (2) can also be derived using other methods, such as a data-driven approach [25].

Thus, according to Eq (3), all analysis results are ultimately compiled into a functional safety analysis matrix:


$$M_{FC}=[\begin{array}{cccc} 1 & 0 & 1 & 0\\ 1 & 0 & 0 & 1\\ 0 & 1 & 0 & 0 \end{array}].$$

According to Eq (4), the functional safety attribute value is obtained as $V_{FC}={\left\|M_{FC}\right\|}_{F}=5$.

#### SOTIF attribute.

Based on the structure of the system abstract model, we analyze its performance insufficiency. In this step, we focus on the typical performance deficiencies in the sensing layer: $Function_{sense}=\{insufficiency_{1},insufficiency_{2},insufficiency_{3}\}$, where *insufficiency*_1_ represents the image resolution limitation affecting distance estimation, *insufficiency*_2_ represents the poor image rendering under low light conditions, and *insufficiency*_3_ represents the misclassification of unexpected/untrained classes.

To identify the mapping relationship described in Eq (5), the mapping between system performance deficiencies and unsafe maneuvers is established through the system’s operational design domain (ODD) boundaries, as shown in in S1 Table (b).

Thus, according to Eq (6), all analysis results are ultimately compiled into a functional safety analysis matrix:


$$M_{SOTIF}=[\begin{array}{ccc} 1 & 0 & 0\\ 1 & 0 & 1\\ 1 & 1 & 0 \end{array}].$$

According to Eq (7), the SOTIF attribute value is obtained as $V_{SOTIF}={\left\|M_{sotif}\right\|}_{F}=5$.

#### Cybersecurity attribute.

Based on the system abstract modeling, a data flow diagram (DFD) [2] is constructed to clarify the information flow process within the system and the information it carries [2]. To demonstrate the analysis process, we only select the “ego speed” information flow for analysis.

To analyze the categories of information threats faced by information flows, Hernan et al. introduced the STRIDE threat model, developed and used by Microsoft in its Security Development Lifecycle (SDL) [23]. STRIDE stands for Spoofing, Tampering, Repudiation, Information disclosure, Denial of service, and Elevation of privilege, representing categories of threats that violate security attributes. By employing the STRIDE framework [26], we identify the categories of cybersecurity attribute violations for information flows. Additionally, using the security guide-word method (SGM) [24], we analyze the connections between threats and failures. More specifically, by identifying hazards through attribute violations and determining triggering causes using security guide-words, we systematically examine the affected components and entry points, identify compromised functionality, and establish the relationship between information attacks and functional faults.

To highlight the main analysis thread in this analysis step, we only analyze the mapping of certain network threats to the functional failures mentioned in the previous steps. The functional relationship between information flows and unsafe maneuvers described in Eq (8) is represented in S1 Table (c).

Thus, according to Eq (10), the cybersecurity matrix is given by


$$M_{CS}=[\begin{array}{cc} 1 & 0\\ 0 & 1\\ 0 & 0 \end{array}].$$

According to Eq (11), the cybersecurity attribute value is $V_{CS}={\left\|M_{CS}\right\|}_{F}=2$. In conclusion, we obtain the final analysis results. According to Eq (12), we have


$$M_{FS}=\left[M_{FC}|M_{SOTIF}|M_{CS}\right]=[\begin{array}{cccccccc} 1 & 0 & 1 & 0 & 1 & 0 & 0 & 0\\ 1 & 0 & 0 & 1 & 1 & 0 & 1 & 1\\ 0 & 1 & 0 & 0 & 1 & 0 & 0 & 0 \end{array}].$$

According to Eqs (13) and (14), *λ* and $V_{FS}$ are calculated as follows:


λ=2,

$$V_{FS}=\lambda \cdot {\left\|M_{FS}\right\|}_{F}=2\cdot 12=24.$$

### Evaluation of triggering coefficient for unsafe maneuvers

Based on the analysis presented in S1 Tables illustrates a summary of the connection between unsafe maneuvers and their triggering causes.

Since obtaining reliable failure rates and the probability of system performance degradation due to triggering conditions involves sensitive design-stage data, which is difficult to access, we have estimated these values using available methods, as this is not the primary focus of the paper. The fault rates are estimated using the method proposed by Wang et al. [18], as follows: the *fault*_1_ rate is $1.93\;\times \;10^{-4}/\text{h}$, the *fault*_2_ rate is $2.00\;\times \;10^{-4}/\text{h}$, the *fault*_3_ rate is $1.00\;\times \;10^{-4}/\text{h}$, and the *fault*_4_ rate is $3.95\;\times \;10^{-4}/\text{h}$. The occurrence rates of the triggering conditions that activate insufficiencies, based on the method outlined in [29], are $3.95\;\times \;10^{-4}/\text{h}$, $1.93\;\times \;10^{-4}/\text{h}$, and $2.32\;\times \;10^{-4}/\text{h}$, respectively.

Software such as TMTe4PT can be used to evaluate the feasibility of network threats based on established threat rules, using the Common Vulnerability Scoring System (CVSS) method [12]. The assessment metrics include the attack vector (AV), attack complexity (AC), privileges required (PR), and user interaction (UI). Thus, the results of the vulnerability evaluation are as follows: 1.23 for $cs_{\text{threat,1}}$ and 2.28 for $cs_{\text{threat,2}}$.

Thus, from the equations above, we obtain the vector of the functional safety risk trigger coefficient, the vector of the SOTIF risk trigger coefficient, and the vector of the cybersecurity risk trigger coefficient. Here, *b* is set to 10. According to Eqs (15), (16), and (17), the risk triggering coefficient vectors are calculated, respectively, as follows:


$$F_{FC}=[\begin{array}{c} 1.45\\ 1.48\\ 0.79\\ 2.16 \end{array}],\;F_{SOTIF}=[\begin{array}{c} 2.16\\ 1.45\\ 1.63 \end{array}],$$

$$VUL=[\begin{array}{c} 1.23\\ 2.28 \end{array}].$$

According to Eq (18), the triggering factor for unsafe maneuvers is:


$$F_{1}=5.41,$$

$$F_{2}=1.96,$$

$$F_{3}=5.09.$$

### Exposure controllability and severity analysis

Due to space limitations, each type of unsafe maneuver analyzed above is associated with a corresponding potentially hazardous scenario. For each scenario, the exposure and severity are determined.

The potential hazardous scenario associated with unsafe acceleration behavior is derived from real-world driving scenarios outlined by the National Highway Traffic Safety Administration (NHTSA) [21], which specifically assesses the performance and safety of the ACC system. The specific scenario for unsafe acceleration is as follows: On curves and exit ramps, with a vehicle ahead, under normal weather conditions. According to this description, the exposure rate for this scenario is E3, and the severity is S3. For unsafe deceleration and unsafe speed maintenance behaviors, potential hazardous scenarios are described in the functional safety analysis of the ACC system using STPA by Xia et al. [20]:

- The potential hazardous scenario for unsafe deceleration behavior is as follows: There is a vehicle behind, on the highway, under normal weather conditions. The exposure rate for this scenario is E4, and the severity is S3.
- The potential hazardous scenario for unsafe speed maintenance behavior is as follows. The vehicle is traveling normally, closely following the vehicle ahead, on the highway, under normal weather conditions. The exposure rate for this scenario is E4, and the severity is also S3.

Based on the previous analysis of system interdependencies, the normal operation of the ACC system relies on the basic functionalities of the central gateway, power system, and brake system, categorizing it as having a highly supportive relationship. Therefore, according to Eq (20), $\beta_{safety}$ =1.47. Given that the ACC system provides clear and concise prompts and requires simple human actions, the human misuse factor can be derived according to Eq (21) as $\beta_{misuse}$ = 1.

Based on the analysis of the above hazardous scenarios, the adjusted controllability for different scenarios is calculated according to Eq (22). In the unsafe deceleration scenario, we assume that the system can control risks by downgrading to a safe mode; hence *c*_1,1_ = 2.94. In the scenario of unsafe acceleration, we assume that the system can enter an emergency mode and operate normally, thus *c^sys^* = 1 and *c*_2,1_ = 1.47. In the scenario of unsafe speed maintenance behavior, human intervention is a must for the vehicle system, but more than 99% ordinary drivers can avoid harm, resulting in *c^sys^* = 3, *c^D^* = 2 and *c*_3,1_ = 6.41.

### Fusion safety risk and contribution factor calculation

Based on the analysis results above, the final evaluation for each unsafe maneuver is carried out according to Eq (23), obtaining the following risk values:


$$risk_{1}=143.21$$

$$risk_{2}=297.83$$

$$risk_{3}=147.30$$

According to Eq (24), the fusion safety risk value is calculated as *risk*_*FS*_ = 588.34.

Subsequently, we calculate the contribution of each safety-related factor according to Eqs (25) and (26):


$$RCF_{fault_{1}}=72.77,\;RCF_{fault_{2}}=28.92,\;RCF_{fault_{3}}=32.55,\;RCF_{fault,4}=40.22,\;RCF_{insufficiency_{1}}=101.69,\;RCF_{insufficiency_{2}}=28.92,\;RCF_{insufficiency_{3}}=40.22.$$

According to Eq (27), the contributions of cyber-threats to the safety risk are calculated as


$$RCF_{CS_{threat,1}}=116.43,$$

$$RCF_{CS_{threat,2}}=130.63.$$

**Fig 5:**
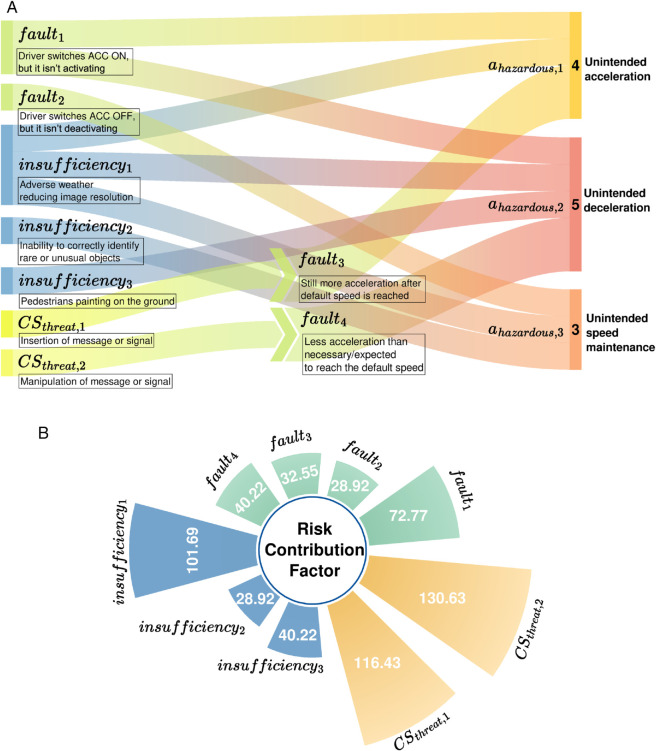
The Over View of Mapping and Risk Contribution Factor. (a) The Sankey diagram maps unsafe maneuvers to their causes, with arrows showing how specific faults, insufficiencies, and cyber threats lead to unsafe maneuvers outcomes. (b) This Radial Bar chart shows the risk contributions of faults, insufficiencies, and cyber threats, with segment sizes reflecting their impact on overall risk.

As illustrated in Fig 5b, cybersecurity issues account for a significant contribution to the total risk. Within these threats, the most severe threat to the safety of system fusion arises from *cs*_*threat*,2_, which involves tampering with the ego speed data transmitted from the powertrain gateway to the engine controller. Consequently, it is vital to strengthen the detection and protection measures for this data stream and its associated information assets.

Among the safety-related factors, the leading contributor to fusion safety risk is *insufficiency*1, identified as the “Image resolution limitation affecting distance estimation.” This problem results in the most significant combined scores for exposure rate, controllability, and severity of hazardous scenarios, highlighting the need to prioritize its performance improvement in the ACC system within this study. In terms of functional safety, *Fault*1 holds the highest contribution to fusion safety risk due to its description as “No activation of the ACC when the ON signal is received.” This indicates that *Fault*1 has the highest risk impact value, necessitating its focus for the enhancement of functional safety design. Since the primary aim of this case study is to validate the framework rather than to produce precise risk metrics, it should be noted that the failure rates, performance insufficiency frequencies, and cybersecurity vulnerability scores in this case study are drawn from published literature without empirical calibration. While suitable for demonstrating the methodological feasibility, they may introduce uncertainty in absolute risk values.

In conclusion, the analysis highlights two key insights in this case study: first, the overall fusion risk value indicates that relying solely on a single safety or security analysis during the design phase is inadequate to address all potential risks, thus requiring a thorough fusion safety evaluation. Moreover, the contributing factors identify *Fault*1 and *cs*_*threat*,2_ within the safety and security domains, which need enhancement to promptly and effectively reduce the overall risk value. Additionally, when dealing with various systems, the fusion risk value serves as a beneficial benchmark for assessing the fusion risk of different systems, aiding in the identification of the crucial system.

## Discussion

Through the case study analysis, we demonstrated the core analytical process and logic of the proposed method. During the modeling phase, we made deliberate trade-offs between modeling precision and analytical complexity by adopting an abstracted system representation. This abstraction retained the essential functional layers and their interactions, ensuring that the model could still capture the true risk characteristics of the system. Importantly, by relying on quantitative fusion metrics rather than subjective judgment, the framework not only identified high-risk elements within individual domains but also uncovered critical cross-domain interactions that are often overlooked in traditional, domain-isolated analyses. These findings underscore the method’s ability to prioritize risks based on their integrated impact, reshaping traditional safety evaluation strategies.

These insights are not limited to the ACC subsystem. With appropriate domain-specific adaptations, the same abstraction principles and analytical logic can be extended to other safety-critical subsystems in ICVs, such as lateral control and perception modules. For each subsystem, system abstraction and inter-layer data flows must be tailored to reflect specific structural and functional characteristics. A deep understanding of system architecture and operational logic is therefore essential to ensure analysis accuracy. While functionally similar subsystems may exist, architectural differences and domain-specific operational patterns can significantly influence the analytical process. These observations highlight the importance of customizing input representations while preserving the consistency of the reasoning logic. In terms of implementation, the framework often relies on historical accident records or expert knowledge to identify hazardous scenarios and failure consequences. However, such information is often sensitive or proprietary, and future extensions may incorporate profiling and data-leakage risks as upstream threat vectors with potential to indirectly trigger unsafe vehicle behaviors and physical harm, enabling their structured integration into the fusion matrix. Studies such as [32–35] provide methodological references for embedding privacy concerns into ICV safety analyses. Consequently, the practical application of the framework must adhere to strict data governance policies to mitigate the risks of data leakage or misuse.

For future validation, we plan to benchmark the proposed framework against established methods such as HARA and STPA on representative systems. A key objective will be to quantify and compare hazard coverage—e.g., the percentage of hazards each method identifies under a shared scenario set—so as to evaluate their respective strengths in capturing multi-domain safety concerns. Furthermore, collaboration with industry stakeholders, including OEMs and Tier-1 suppliers, will be essential to test practical feasibility and incorporate domain-specific feedback. This iterative feedback loop will be critical to maturing the framework for real-world adoption and integration into ICV safety engineering workflows.

## Conclusion

This study presents a unified analytical framework for ICV safety that integrates functional safety, SOTIF, and cybersecurity by linking diverse risks to vehicle-level unsafe behaviors. Using a backpropagation-like approach, it traces the impact of safety issues on overall risk and prioritizes mitigation efforts based on safety gradients. In the ACC case study, the framework effectively identified and quantified critical safety risks across multiple domains. It revealed specific weak points—such as *cs*_*threat*,2_, which exhibited the highest RCF of 130.63—and demonstrated how the proposed metrics can support targeted optimization during the product design phase. However, the framework relies on sensitive inputs, including detailed system architecture and historical accident data. Its current form is conceptual and requires empirical validation. Additionally, dedicated tools for scenario mapping, risk matrix construction, and multi-domain data integration are necessary for practical deployment. In summary, to address the complex, integrated safety risks associated with ICVs, this paper proposes a systematic analytical framework that offers a new perspective on system safety. It fills a critical gap in existing methods by enabling unified, quantitative assessment of functional safety, SOTIF, and cybersecurity—domains typically addressed in isolation—and thus contributes to the advancement of intelligent and connected vehicles.

## Supporting information

S1 Table Supplementary tables for the case study. (a) Mapping relationship between faults and hazardous maneuvers. (b) Mapping relationship between insufficiencies and hazardous maneuvers. (c) Cyber threats and vulnerabilities analysis related to ego speed data flow.
(PDF)
