# Supplementary material for: A fusion safety and security analysis framework for intelligent and connected vehicles
Source: PLoS One. 2025 Sep 22;20(9):e0332050. doi: 10.1371/journal.pone.0332050 (PMC12453215; doi:10.1371/journal.pone.0332050)
Supplement: S1 Table — (a) Mapping relationship between faults and hazardous maneuvers. (b) Mapping relationship between insufficiencies and hazardous maneuvers. (c) Cyber threats and vulnerabilities analysis related to ego speed data flow. (PDF) [file pone.0332050.s001.pdf]

Fault-ID	Technical cause	Hazardous maneuver
$fault_1$	Driver switches ACC ON, but it isn't activating	$a_{\text{hazardous},1}$ $a_{\text{hazardous},2}$
$fault_2$	Driver switches ACC OFF, but it isn't deactivating	$a_{\text{hazardous},3}$
$fault_3$	Still more acceleration after default speed is reached	$a_{\text{hazardous},1}$
$fault_4$	Less acceleration than necessary/expected to reach the default speed	$a_{\text{hazardous},2}$

(a) Mapping relationship between faults and hazardous maneuvers. This table describes the mapping relationship between identified faults in the system, and the corresponding hazardous maneuvers. "Technical cause" describes the underlying issue causing the fault, while "Hazardous maneuver" maps the fault to the unsafe maneuver on vehicle level.

Insufficiency-ID	Triggering condition	Unsafe maneuver
$insufficiency_1$	Adverse weather conditions cause camera image blur, affecting distance estimation	$a_{\text{hazardous},1}$ $a_{\text{hazardous},2}$ $a_{\text{hazardous},3}$
$insufficiency_2$	Inability to correctly identify rare or unusual objects	$a_{\text{hazardous},3}$
$insufficiency_3$	Pedestrians painting on the ground	$a_{\text{hazardous},2}$

(b) Mapping relationship between insufficiencies and hazardous maneuvers. This table illustrates the mapping relationship between system insufficiencies with their triggering conditions, and the resulting unsafe maneuvers. "Insufficiency-ID" represents specific insufficiencies in the system. "Triggering condition" describes the situations or events that activate these insufficiencies. "Unsafe maneuver" identifies the corresponding hazardous actions.

Data flow	Threat-ID	STRIDE					Can be triggered by	Component	Entry point	Name function	Fault-ID
		S	T	R	I	D					
Ego speed	$CS_{\text{threat},1}$	X					Insertion of message or signal	Engine control	Powertrain gateway	Accelerate over default speed	$f_{\text{fault},3}$
	$CS_{\text{threat},2}$		X				Manipulation of message or signal	Engine control	Powertrain gateway	Decelerate to default speed	$f_{\text{fault},4}$

(c) Cyber Threats and Vulnerabilities Analysis. This table presents an analysis of the cyber threats and vulnerabilities associated with the data flow of ego speed. The "Threat-ID" column identifies specific threats, categorized using the STRIDE framework.Columns 4, 5, 6, and 7 are based on templates derived from the SGD method and outline the analysis process of the relationship between information attacks and functional faults. The "Can be triggered by" column describes the potential causes of each threat, while the "Component or system" and "Entry point" columns indicate the affected system and the point of entry for the threat. The 'Name function' column maps the threat to a corresponding function fault, with a designated "Fault-ID".

Table 1: Supplementary Tables for the Case Study. (a) Faults and hazardous maneuvers. (b) Insufficiencies and hazardous maneuvers. (c) Cyber threat analysis.
